# Supplementary material for: A Comparison of Statistical Methods for Time‐To‐Event Analyses in Randomized Controlled Trials Under Non‐Proportional Hazards
Source: Stat Med. 2025 Feb 20;44(5):e70019. doi: 10.1002/sim.70019 (PMC11840476; doi:10.1002/sim.70019)
Supplement: Supplementary file 1 — Data S1. Supplementary Figures. [file SIM-44-0-s001.pdf]

Supplementary material to "A comparison of statistical methods for time-to-event analyses in randomized controlled trials under non-proportional hazards"

Florian Klinglmüller[1], Tobias Fellinger[1], Franz König[2], Tim Friede[3], Andrew C. Hooker[4], Harald Heinzl[2], Martina Mittlböck[2], Jonas Brugger[2,3], Maximilian Bardo[3], Cynthia Huber[3], Norbert Benda[3,5], Martin Posch[2,*], and Robin Ristl[2]

[1]Austrian Agency for Health and Food Safety, Vienna, Austria
[2]Medical University of Vienna, Center for Medical Data Science, Vienna, Austria
[3]University Medical Center Göttingen, Department of Medical Statistics, Göttingen, Germany
[4]Dept. of Pharmacy, Uppsala University, Uppsala, Sweden
[5]Federal Institute for Drugs and Medical Devices (BfArM), Research Division, Bonn, Germany
[*]Corresponding author, e-mail: martin.posch@meduniwien.ac.at

December 4, 2024

1 Supplementary Figures

The supplementary Figures S1 to S7 show the survival functions, hazard functions and hazard ratio functions for all simulation scenarios considered in the paper. Details regarding the parameter values in the simulation scenarios are found in Section 3 of the main paper. The supplementary Figures S8 to S10 illustrate how the power of tests for milestone survival differences, RMST differences and average hazard ratio changes with increasing milestone/cut-off time in the considered scenarios. The supplementary Figures S11 to S13 show the coverage probability of nominal 95% confidence intervals for milestone survival differences, RMST differences and average hazard ratio changes with increasing milestone/cut-off time in the considered scenarios.

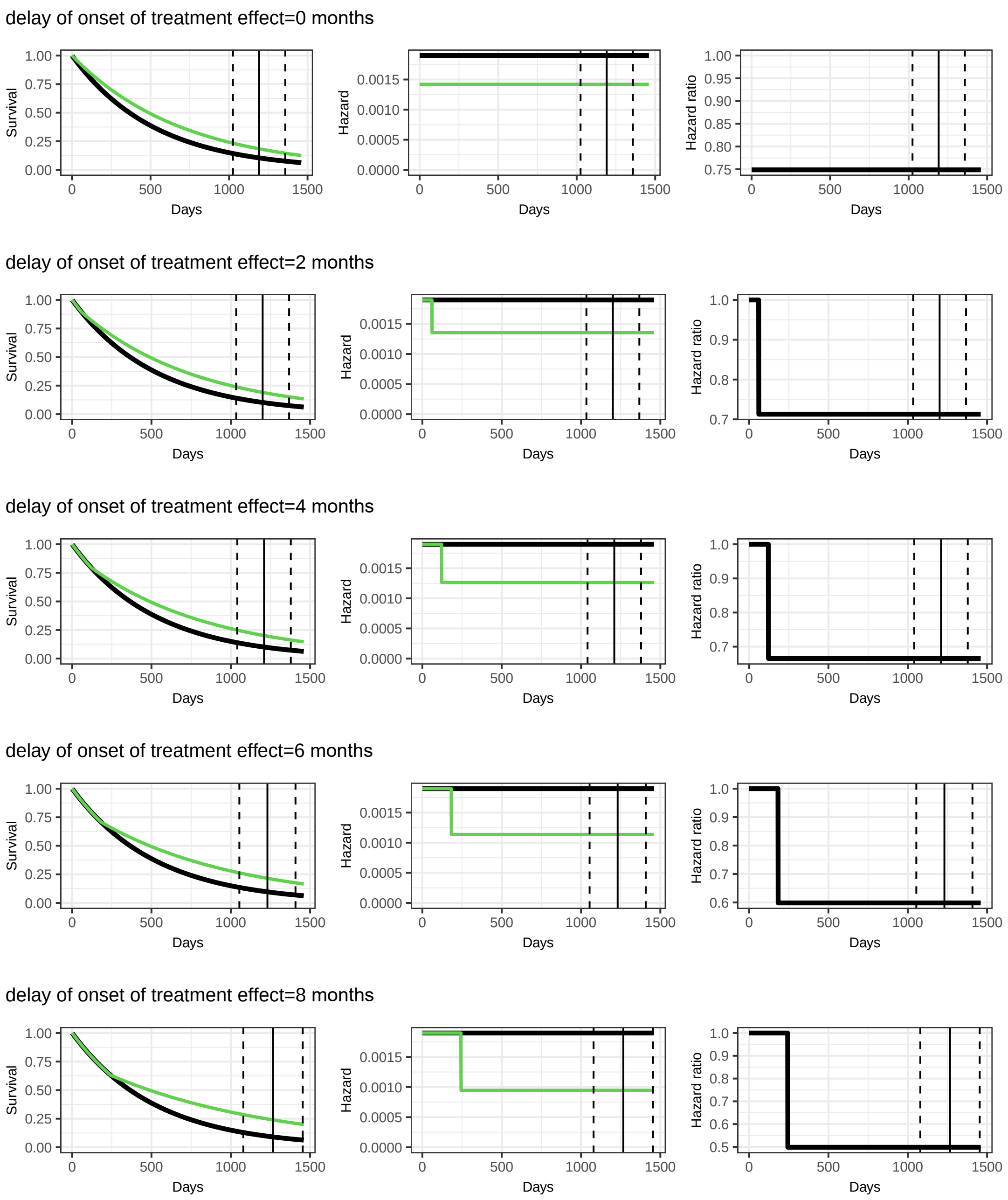

Figure S1: Survival functions, hazard functions and hazard ratio functions in the scenarios with delayed onset of treatment effect. Each row of the figure represents a scenario with a specific delay time as indicated in the superscripts. The left panel shows survival functions for treatment (green) and control (black). In each row, the center panel shows the corresponding hazard functions. The right panel shoes the hazard ratio function. Additionally, the distribution of the follow-up time across simulation runs is shown in terms of the mean follow-up time, represented by a vertical solid line, and mean +/- 2 standard deviations, represented by vertical dashed lines.

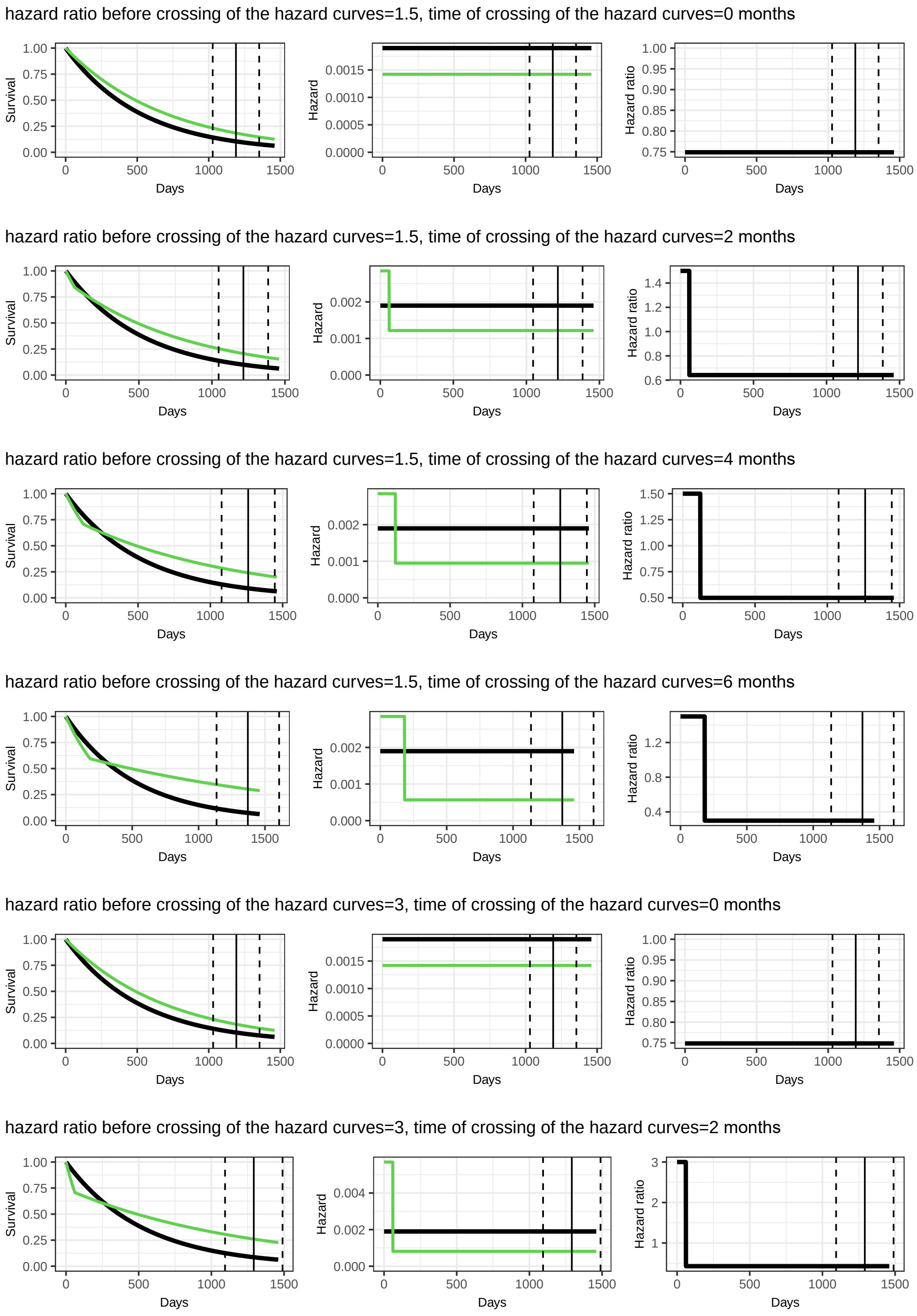

Figure S2: Survival functions, hazard functions and hazard ratio functions in the scenarios with crossing hazards. Each row of the figure represents a scenario with a specific crossing time as indicated in the superscripts. The left panel shows survival functions for treatment (green) and control (black). In each row, the center panel shows the corresponding hazard functions. The right panel shoes the hazard ratio function. Additionally, the distribution of the follow-up time across simulation runs is shown in terms of the mean follow-up time, represented by a vertical solid line, and mean +/- 2 standard deviations, represented by vertical dashed lines.

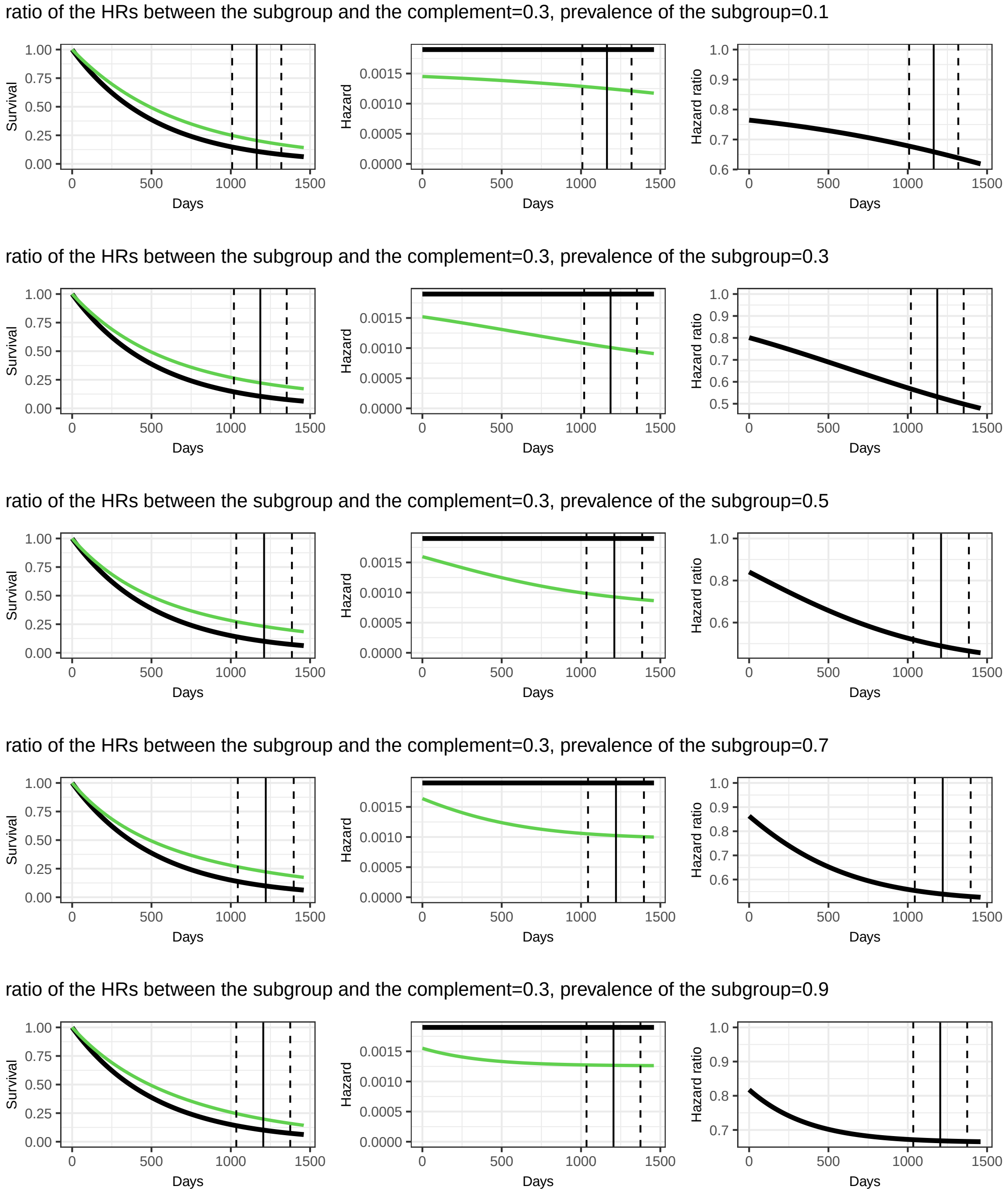

Figure S3: Survival functions, hazard functions and hazard ratio functions in the scenarios with biomarker subgroups. Each row of the figure represents a scenario with a specific hazard ratio between biomarker positive and negative patients and specific prevalence of biomarker positive patients as indicated in the superscripts. The left panel shows survival functions for treatment (green) and control (black). In each row, the center panel shows the corresponding hazard functions. The right panel shoes the hazard ratio function. Additionally, the distribution of the follow-up time across simulation runs is shown in terms of the mean follow-up time, represented by a vertical solid line, and mean +/- 2 standard deviations, represented by vertical dashed lines.

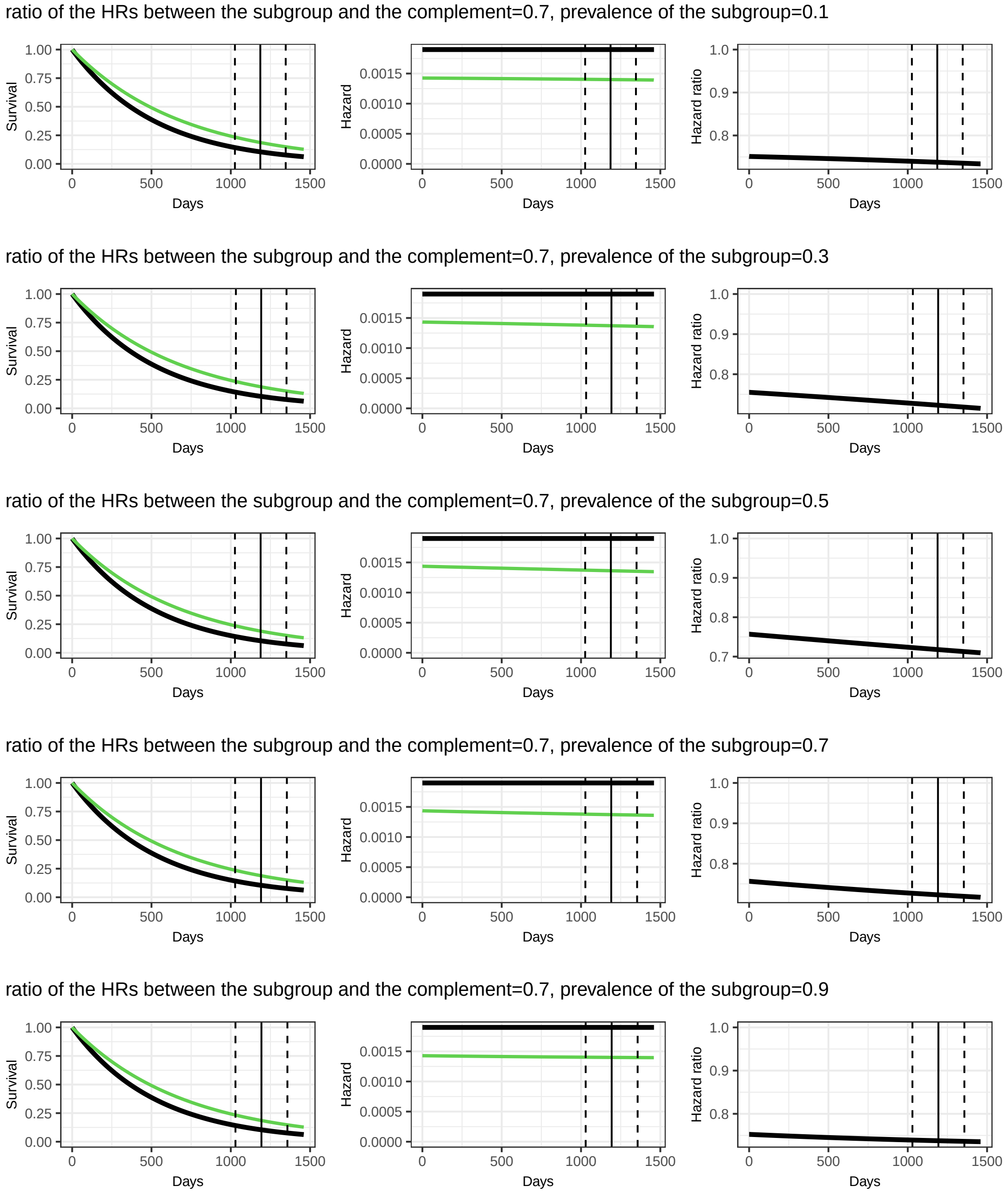

Figure S4: Continued: Survival functions, hazard functions and hazard ratio functions in the scenarios with biomarker subgroups. Each row of the figure represents a scenario with a specific hazard ratio between biomarker positive and negative patients and specific prevalence of biomarker positive patients as indicated in the superscripts. The left panel shows survival functions for treatment (green) and control (black). In each row, the center panel shows the corresponding hazard functions. The right panel shoes the hazard ratio function. Additionally, the distribution of the follow-up time across simulation runs is shown in terms of the mean follow-up time, represented by a vertical solid line, and mean +/- 2 standard deviations, represented by vertical dashed lines.

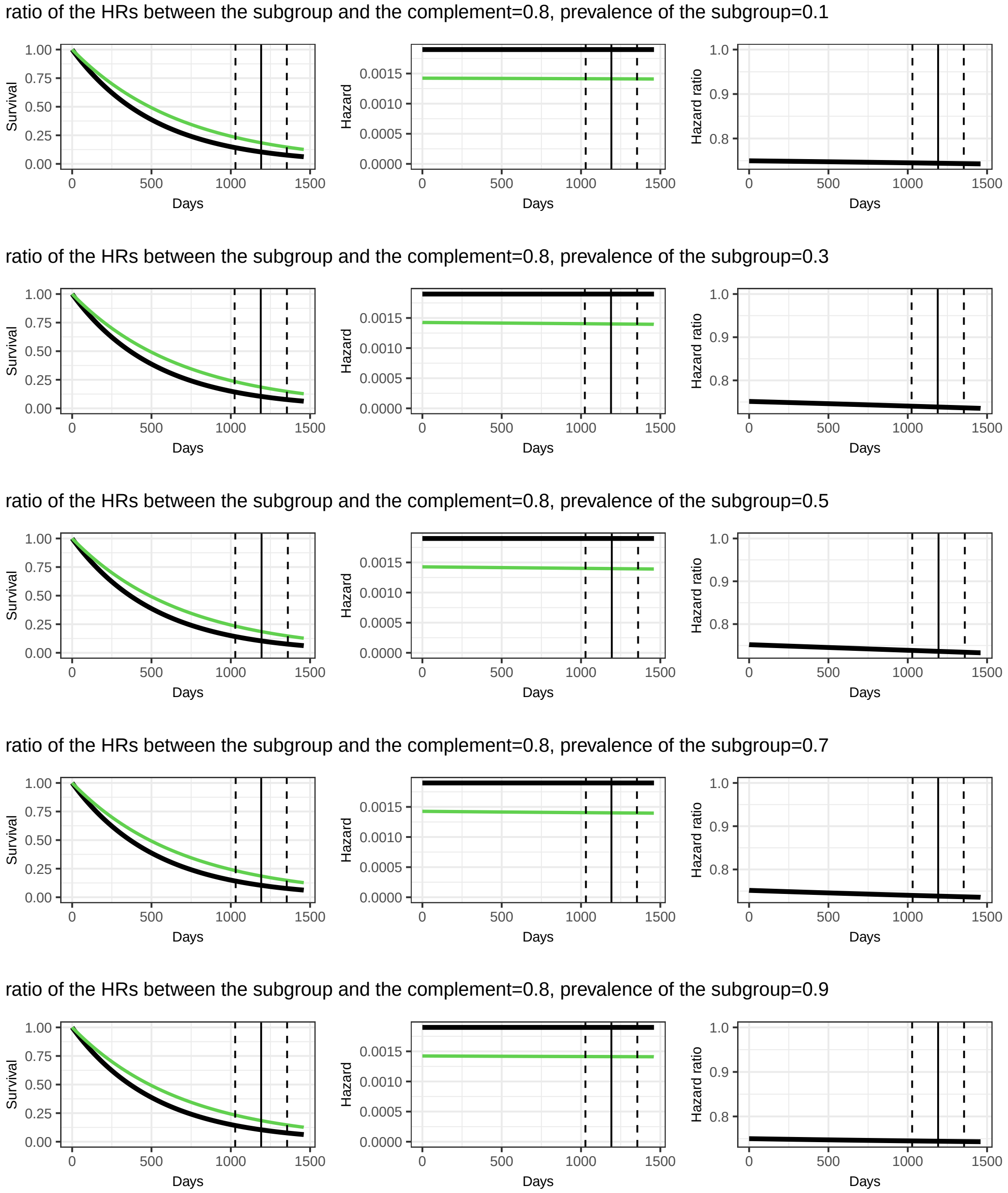

Figure S5: Continued: Survival functions, hazard functions and hazard ratio functions in the scenarios with biomarker subgroups. Each row of the figure represents a scenario with a specific hazard ratio between biomarker positive and negative patients and specific prevalence of biomarker positive patients as indicated in the superscripts. The left panel shows survival functions for treatment (green) and control (black). In each row, the center panel shows the corresponding hazard functions. The right panel shoes the hazard ratio function. Additionally, the distribution of the follow-up time across simulation runs is shown in terms of the mean follow-up time, represented by a vertical solid line, and mean +/- 2 standard deviations, represented by vertical dashed lines.

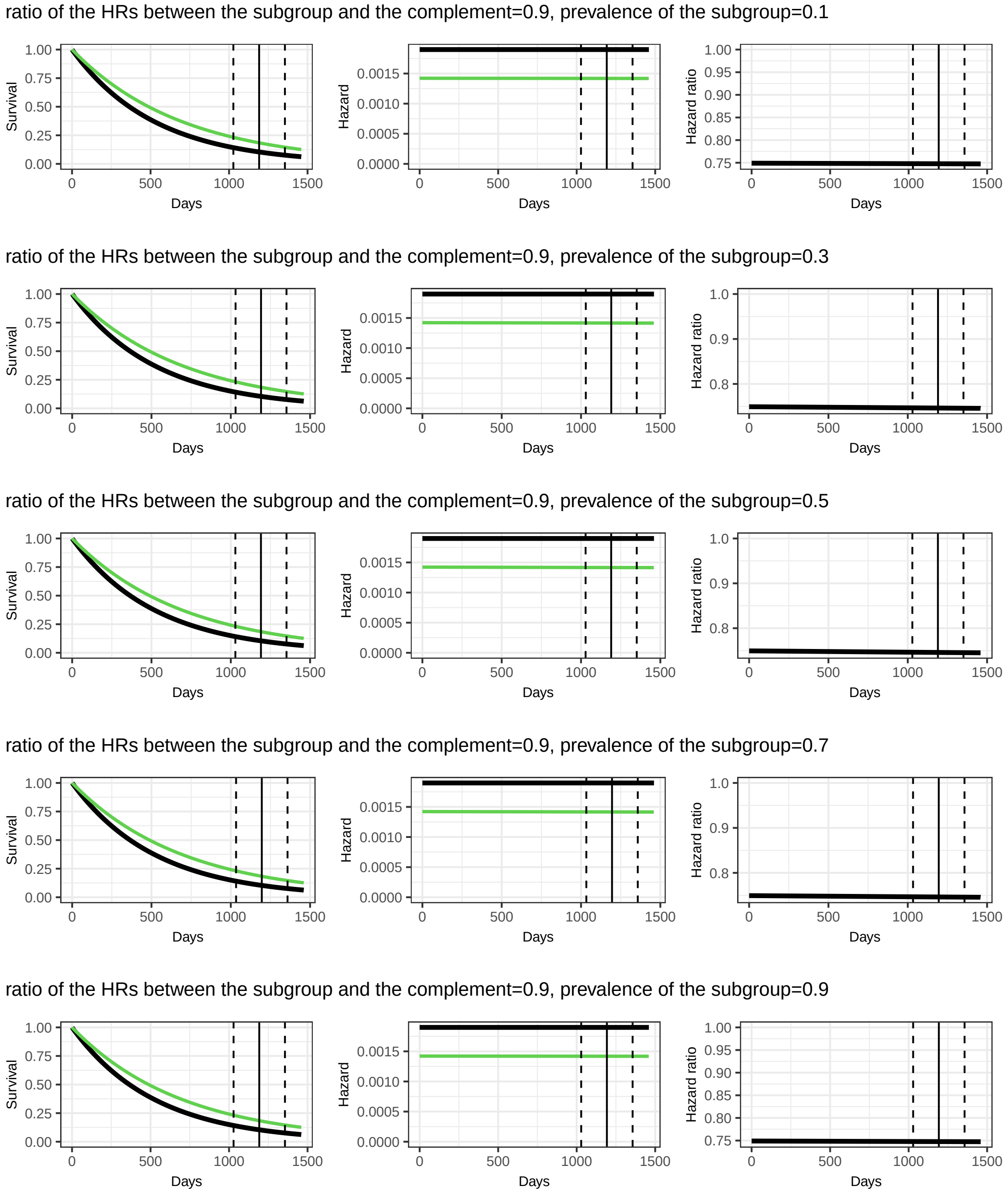

Figure S6: Continued: Survival functions, hazard functions and hazard ratio functions in the scenarios with biomarker subgroups. Each row of the figure represents a scenario with a specific hazard ratio between biomarker positive and negative patients and specific prevalence of biomarker positive patients as indicated in the superscripts. The left panel shows survival functions for treatment (green) and control (black). In each row, the center panel shows the corresponding hazard functions. The right panel shoes the hazard ratio function. Additionally, the distribution of the follow-up time across simulation runs is shown in terms of the mean follow-up time, represented by a vertical solid line, and mean +/- 2 standard deviations, represented by vertical dashed lines.

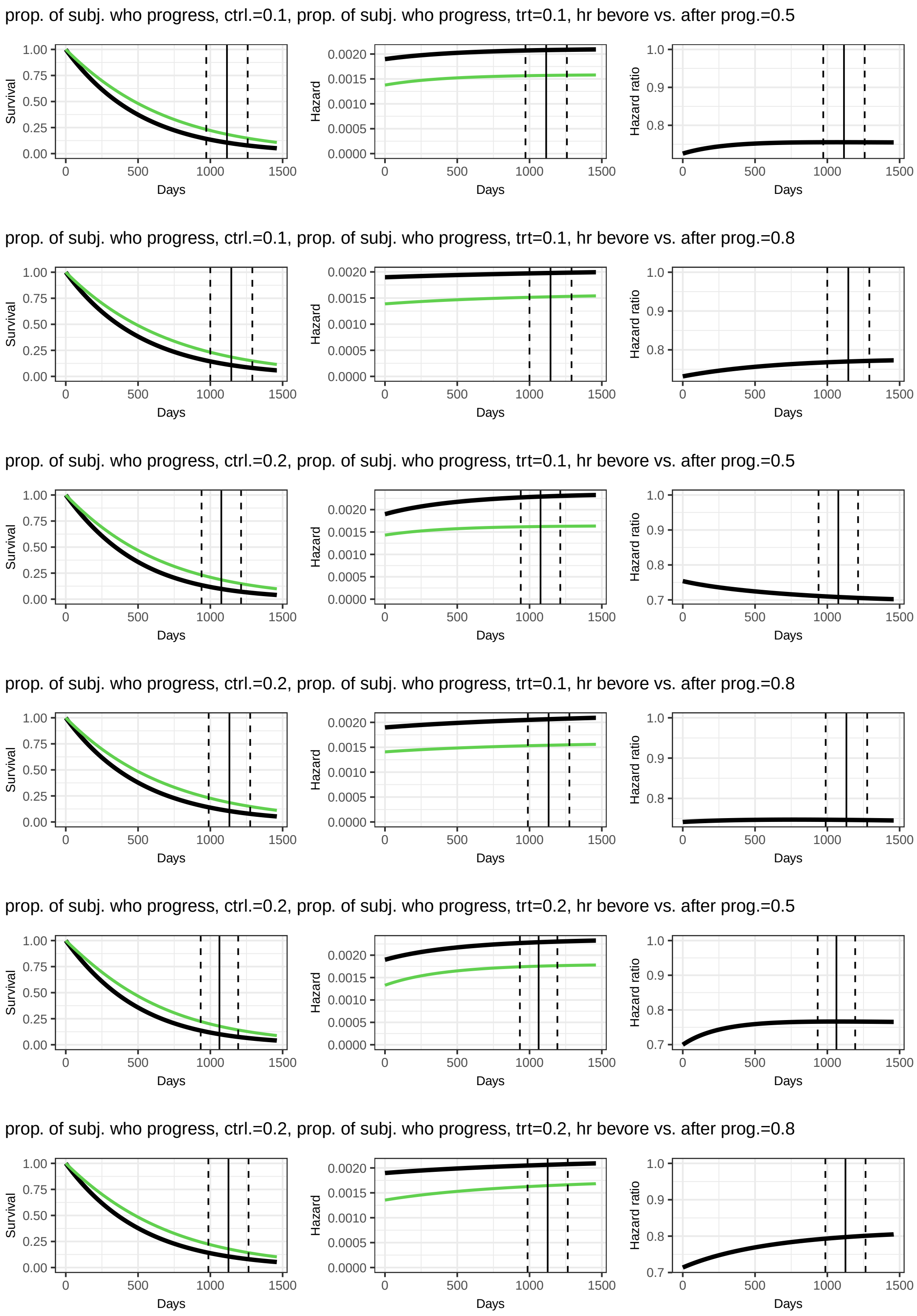

Figure S7: Survival functions, hazard functions and hazard ratio functions in the scenarios with disease progression. Each row of the figure represents a scenario with values for the proportion of subjects who progress under treatment and control and the hazard ratio before versus after progression as indicated in the superscripts. The left panel shows survival functions for treatment (green) and control (black). In each row, the center panel shows the corresponding hazard functions. The right panel shoes the hazard ratio function. Additionally, the distribution of the follow-up time across simulation runs is shown in terms of the mean follow-up time, represented by a vertical solid line, and mean +/- 2 standard deviations, represented by vertical dashed lines.

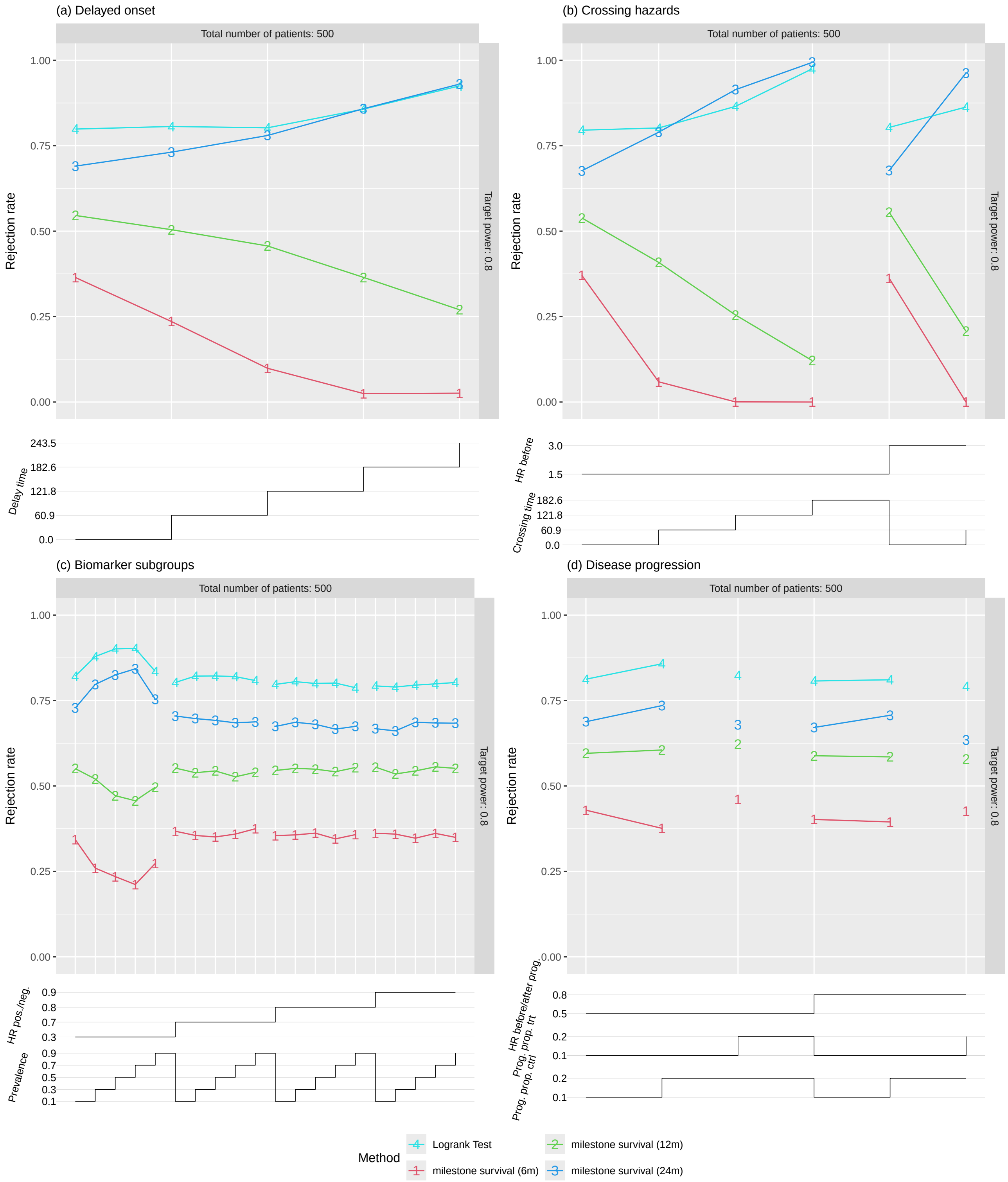

Figure S8: Power of hypothesis tests for the difference in 6, 12 and 24 month survival probabilities and the unweighted logrank test in the scenarios with (a) delayed onset of treatment effect, (b) crossing hazard, (c) biomarker subgroups and (d) disease progression.

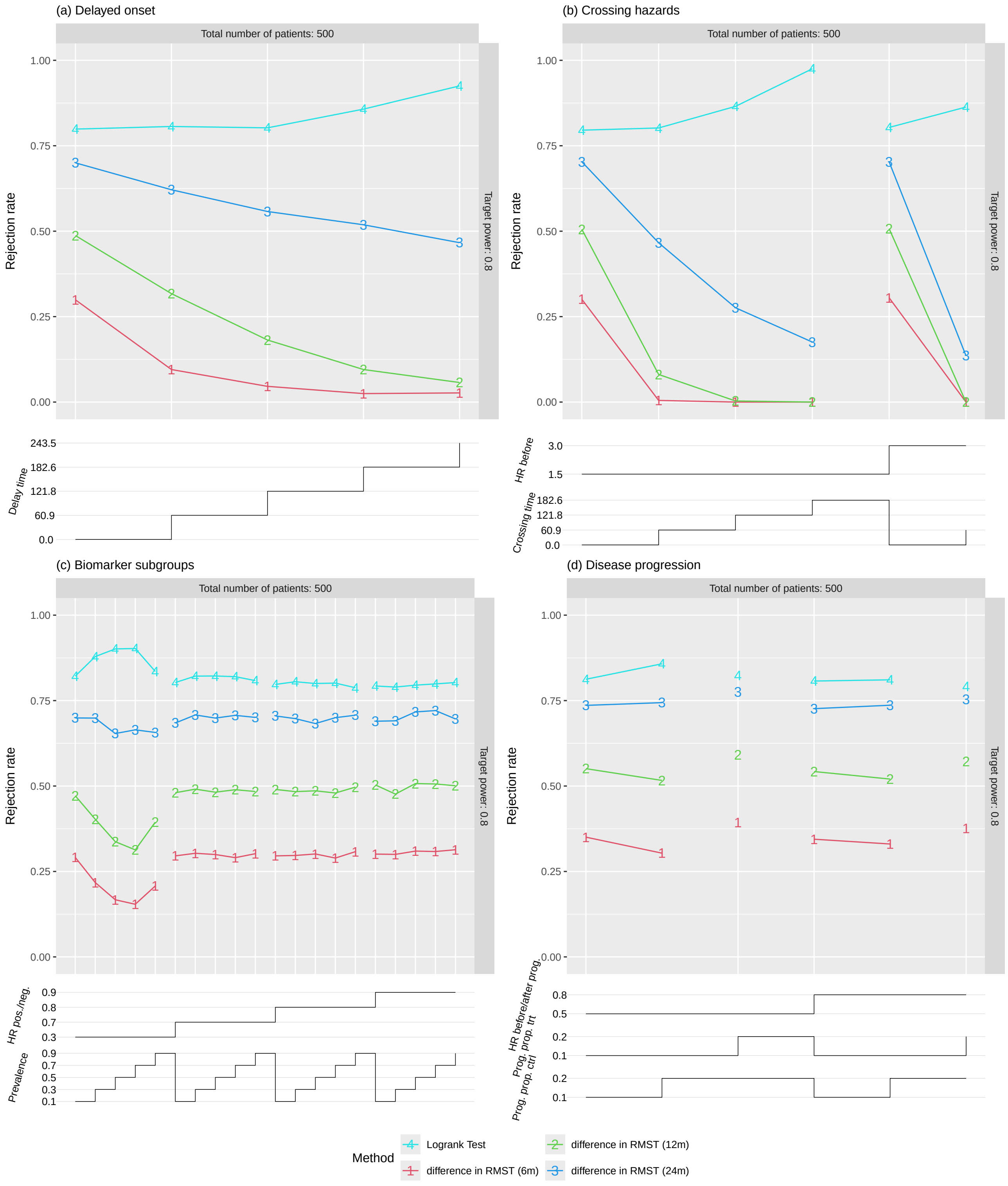

Figure S9: Power of hypothesis tests for the difference in restricted mean survival time (RMST) with cut-off at 6, 12 and 24 months and the unweighted logrank test in the scenarios with (a) delayed onset of treatment effect, (b) crossing hazard, (c) biomarker subgroups and (d) disease progression.

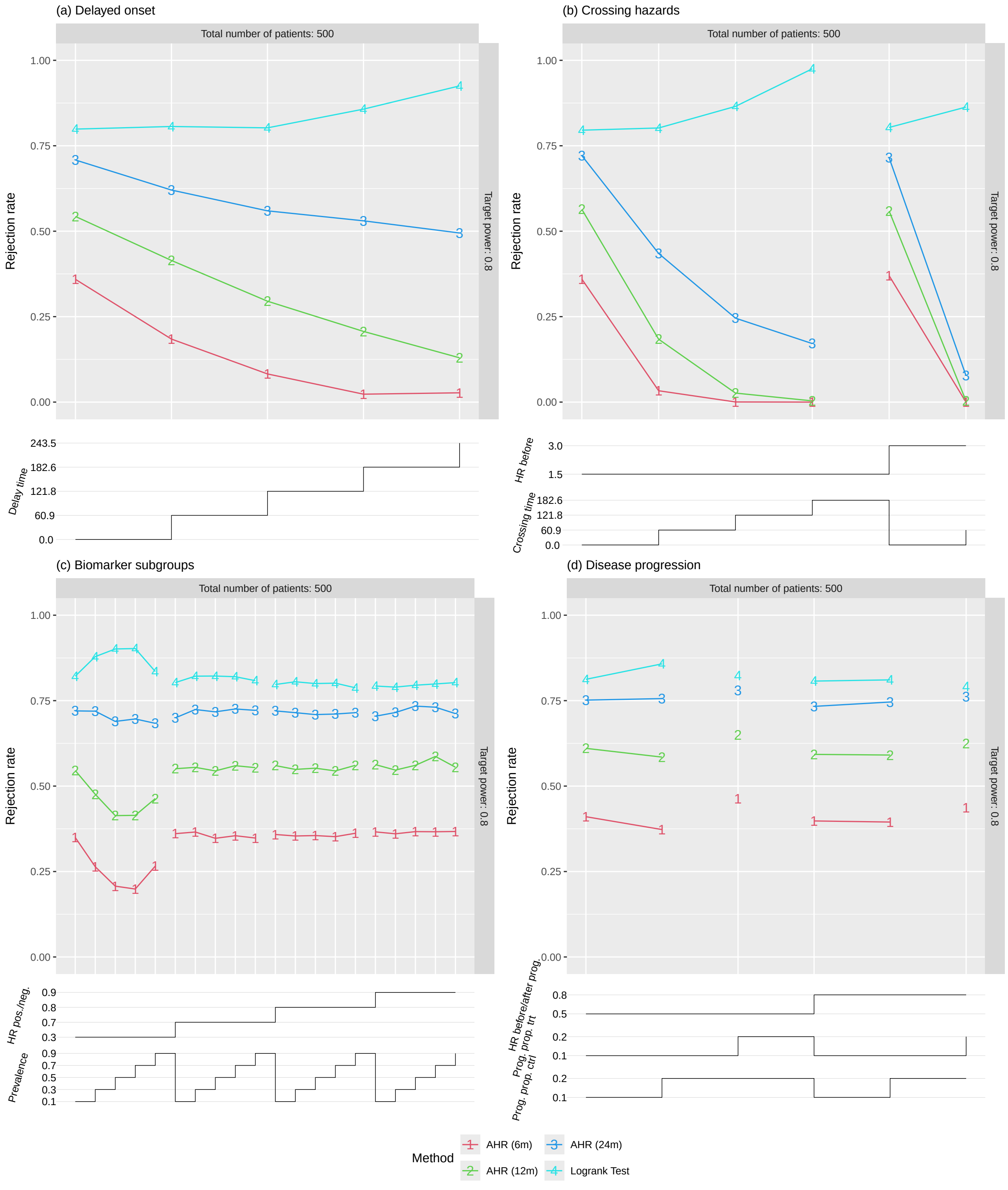

Figure S10: Power of hypothesis tests for the average hazard ratio (AHR) with cut-off at 6, 12 and 24 months and the unweighted logrank test in the scenarios with (a) delayed onset of treatment effect, (b) crossing hazard, (c) biomarker subgroups and (d) disease progression.

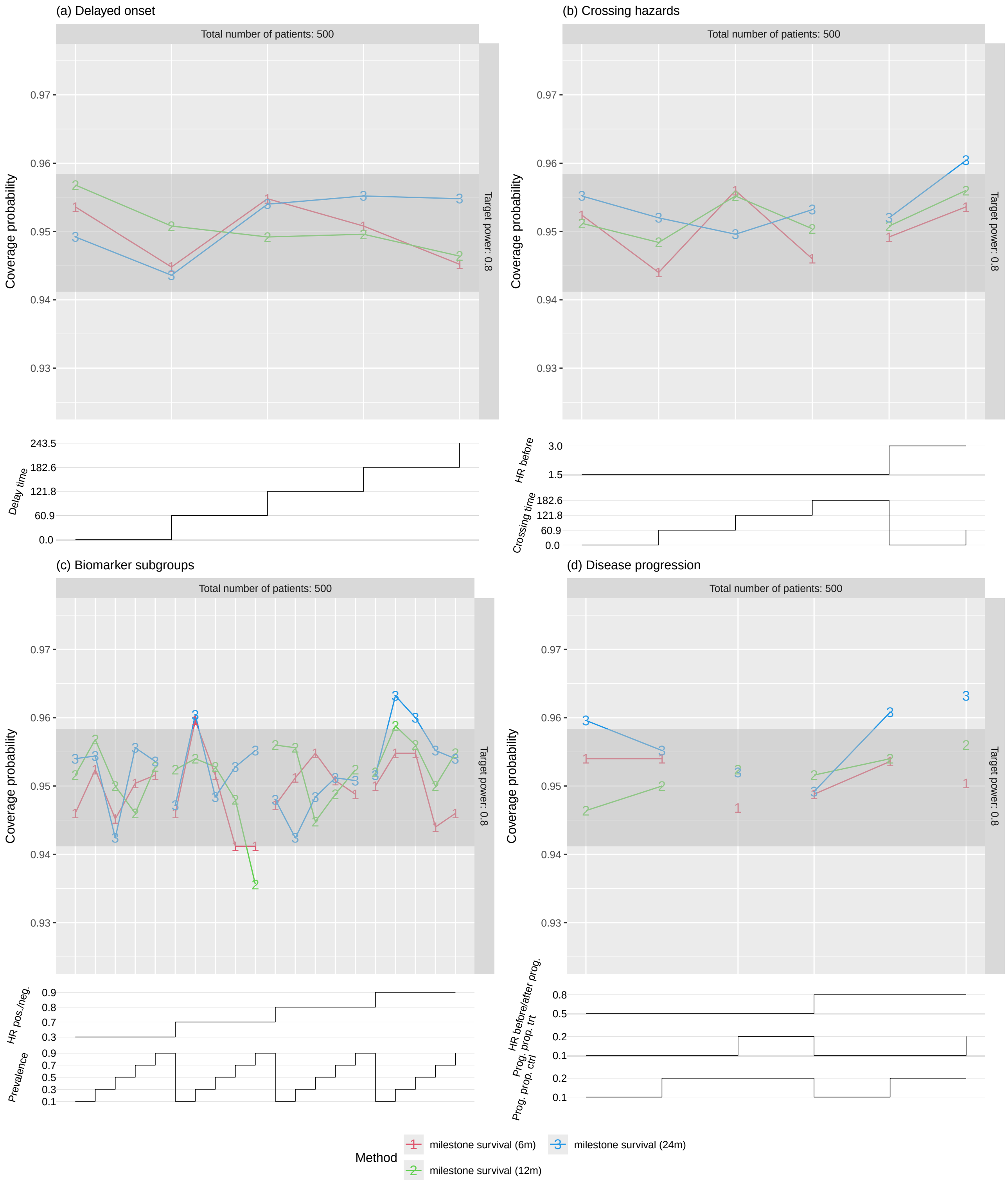

Figure S11: Coverage probability of nominal 95% confidence intervals for the difference in 6, 12 and 24 month survival probabilities in the scenarios with (a) delayed onset of treatment effect, (b) crossing hazard, (c) biomarker subgroups and (d) disease progression. The dark grey regions indicate the nominal coverage probability of 95% $\pm$ 1.96 times the simulation standard error for an interval with exactly 95% coverage.

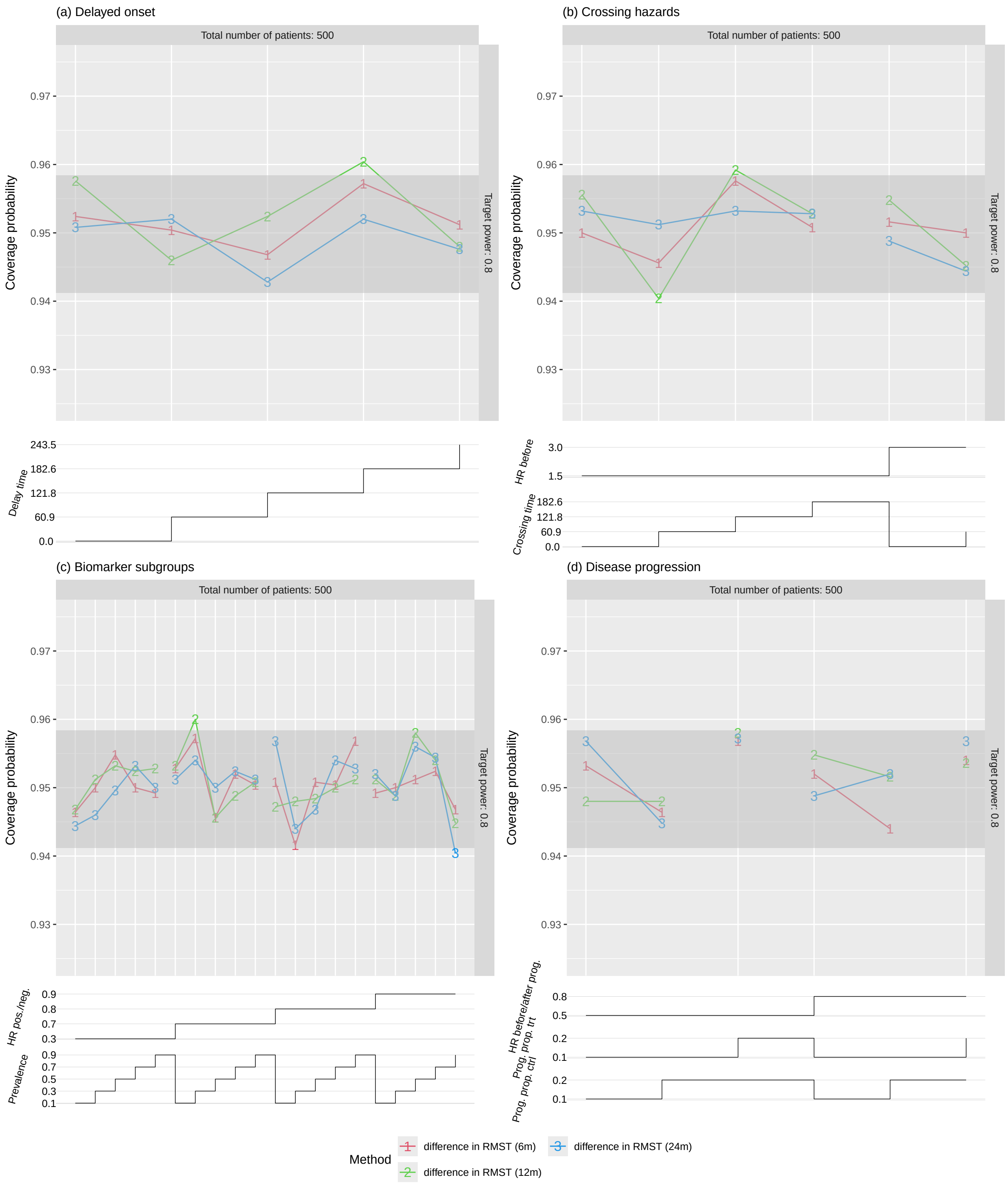

Figure S12: Coverage probability of nominal 95% confidence intervals for the difference in restricted mean survival time (RMST) with cut-off at 6, 12 and 24 months in the scenarios with (a) delayed onset of treatment effect, (b) crossing hazard, (c) biomarker subgroups and (d) disease progression. The dark grey regions indicate the nominal coverage probability of 95% ± 1.96 times the simulation standard error for an interval with exactly 95% coverage.

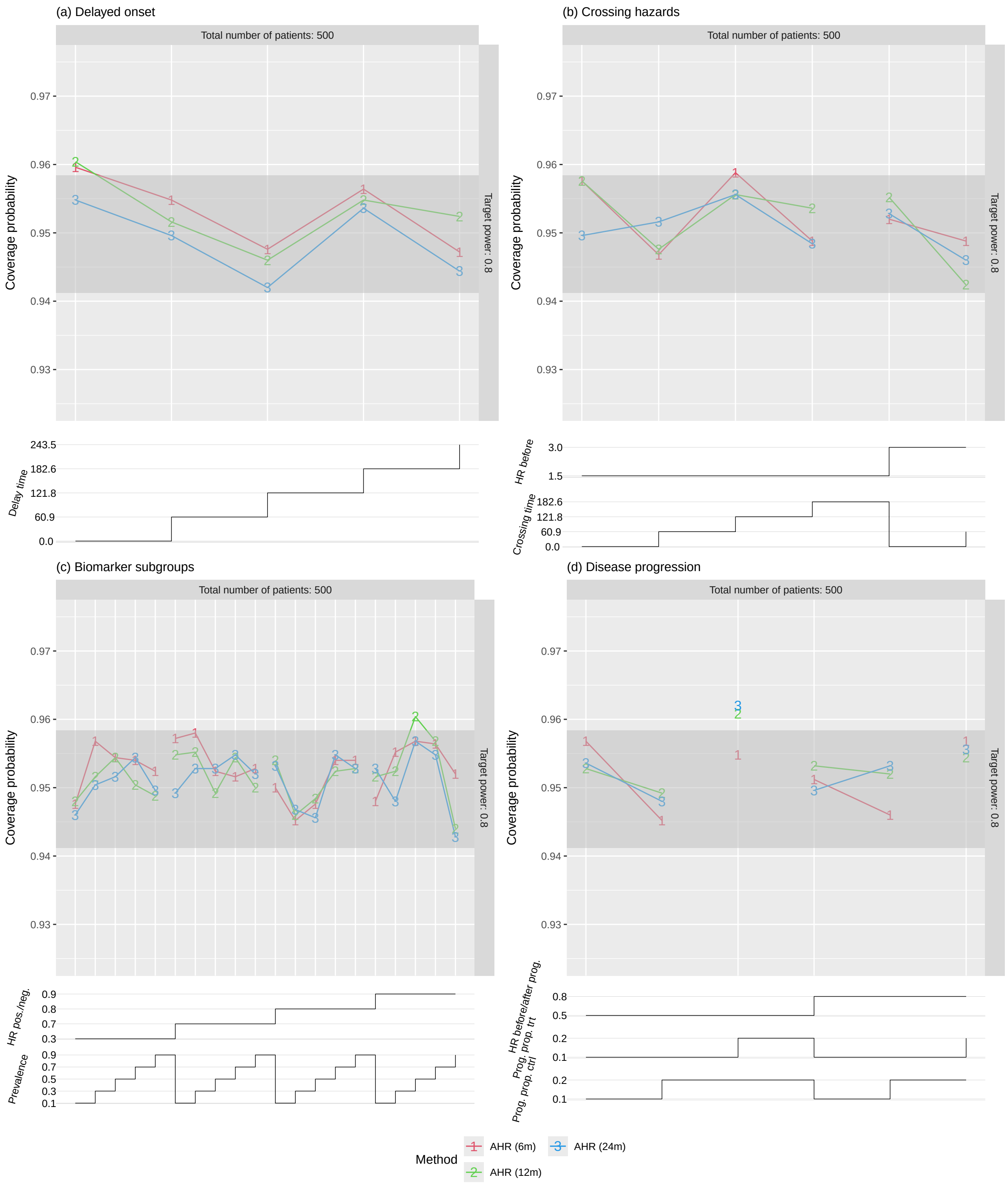

Figure S13: Coverage probability of nominal 95% confidence intervals for the average hazard ratio (AHR) with cut-off at 6, 12 and 24 months in the scenarios with (a) delayed onset of treatment effect, (b) crossing hazard, (c) biomarker subgroups and (d) disease progression. The dark grey regions indicate the nominal coverage probability of 95% ± 1.96 times the simulation standard error for an interval with exactly 95% coverage.

2 Exemplary R code to calculate true parameter values

The following R code includes a minimal running example of how the true parameter values for the average hazard ratio and the restricted mean survival times were calculated based on the assumed true survival functions and using numeric integration.

```
# Calculation of the True Summary Statistics
#
# Example for a scenario with delayed onset of treatment effect after 8 months,
# median survival in the control arm 12 months 500 patients and
# effect size under PH 0.8.
#
# This script is an excerpt of the function fast_real_statistics implemented in
# the R package SimNPH. The full code used in the simulation study can be
# accessed here:
# https://github.com/SimNPH/SimNPH/blob/3485630a94731b258229d0647cb1238394dcd5f8/R/internal_real_stat
#
# haz_trt     hazard function of the treatment arm
# pdf_trt     probability density function of the treatment arm
# surv_trt    survival function of the treatment arm
# haz_ctrl    hazard function of the control arm
# pdf_ctrl    probability density function of the control arm
# surv_ctrl   survival function of the control arm
# N_trt       number of patients in the treatment arm
# N_ctrl      number of patients in the control arm
# cutoff      cutoff used to calculate rmst and average hazard ratios

install.packages("SimNPH")
install.packages("miniPCH")
library(SimNPH)
library(miniPCH)


# define parameters for example scenario ----------------------------------

functions_trt <- pch_functions(
  t      = c(0, m2d(8)),
  lambda = c(m2r(12), 0.000945716047185455)
)

functions_ctrl <- pch_functions(
  t      = c(0),
  lambda = c(m2r(12))
)

haz_trt     <-  functions_trt$h
pdf_trt     <-  functions_trt$d
surv_trt    <-  functions_trt$s
haz_ctrl    <- functions_ctrl$h
pdf_ctrl    <- functions_ctrl$d
surv_ctrl   <- functions_ctrl$s
N_trt       <- 250
N_ctrl      <- 250
cutoff      <- m2d(12)


# calculation of true summary statistics ----------------------------------

# define helper functions for the calculation of the average hazard ratios
h  <- \(t){haz_trt(t)+haz_ctrl(t)}
```

```
f  <- \(t){(1/(N_trt+N_ctrl))*(N_trt*pdf_trt(t) + N_ctrl*pdf_ctrl(t))}

# rectangle rule (more robust in some settings where integrate fails)
myint<-function(f,lower,upper,steps=1000) {
  delta<-(upper-lower)/steps
  x<-seq(lower+delta/2,upper-delta/2,delta)
  sum(f(x)*delta)
}

#AHR
true_avg_HR_fun0<-function(x) surv_ctrl(x)*pdf_trt(x)
true_avg_HR_fun1<-function(x) surv_trt(x)*pdf_ctrl(x)
#integrate can run into numeric problems with NPH objects, myint is more robust
Int0<-myint(true_avg_HR_fun0,lower=0,upper=cutoff)
Int1<-myint(true_avg_HR_fun1,lower=0,upper=cutoff)
# Average Hazard Ratio calculated by rectangle rule
AHR_myint<-Int0/Int1
print(AHR_myint)

Int0A<-integrate(true_avg_HR_fun0,lower=0,upper=cutoff)
Int1A<-integrate(true_avg_HR_fun1,lower=0,upper=cutoff)
# Average Hazard Ratio calculated with integrate function
AHR_integrate<-Int0A$value/Int1A$value
print(AHR_integrate)

# RMST Treatment
rmst_trt = integrate(surv_trt, 0, cutoff)$value
print(rmst_trt)
# RMST Control
rmst_ctrl = integrate(surv_ctrl, 0, cutoff)$value
print(rmst_ctrl)
```
